# Congenital deafness is associated with specific somatosensory deficits in adolescents

**DOI:** 10.1038/s41598-017-04074-0

**Published:** 2017-06-26

**Authors:** Rabih Moshourab, Valérie Bégay, Christiane Wetzel, Jan Walcher, Steven Middleton, Manfred Gross, Gary R. Lewin

**Affiliations:** 1 Department of Neuroscience, Max-Delbrück Center for Molecular Medicine, Robert-Rössle Straße 10, D-13092 Berlin, Germany; 2 Department of Anesthesiology and Operative Intensive Care Medicine, Campus Virchow Klinikum and Campus Mitte, Charité - Universitätsmedizin Berlin, Augustenburger Platz 1, 13353 Berlin, Germany; 3 Klinik für Audiologie und Phoniatrie, Campus Virchow Klinikum, Augustenburger Platz 1, 13353 Berlin, Germany; 4 Neuroscience Research Center and Cluster of Excellence NeuroCure, Charité-Universitätsmedizin, Berlin, Germany

## Abstract

Hearing and touch represent two distinct sensory systems that both rely on the transformation of mechanical force into electrical signals. Here we used a battery of quantitative sensory tests to probe touch, thermal and pain sensitivity in a young control population (14–20 years old) compared to age-matched individuals with congenital hearing loss. Sensory testing was performed on the dominant hand of 111 individuals with normal hearing and 36 with congenital hearing loss. Subjects with congenital deafness were characterized by significantly higher vibration detection thresholds at 10 Hz (2-fold increase, P < 0.001) and 125 Hz (P < 0.05) compared to controls. These sensory changes were not accompanied by any major change in measures of pain perception. We also observed a highly significant reduction (30% compared to controls p < 0.001) in the ability of hearing impaired individual’s ability to detect cooling which was not accompanied by changes in warm detection. At least 60% of children with non-syndromic hearing loss showed very significant loss of vibration detection ability (at 10 Hz) compared to age-matched controls. We thus propose that many pathogenic mutations that cause childhood onset deafness may also play a role in the development or functional maintenance of somatic mechanoreceptors.

## Introduction

The ability of humans to perceive different modalities of cutaneous sensory stimuli can be accurately assessed using quantitative sensory testing. Indeed, quantitative sensory testing is an integral part of the clinical examination in patients with sensory disorders including pain^1–4^. Psychophysical testing reveals that human performance in specific sensory tasks is in part determined by the sensitivity of the sensory receptors activated by the stimulus^5–7^. The skin is equipped with a wide variety of mechanoreceptors and nociceptors that are tuned to respond optimally to different qualities of mechanical or thermal stimuli^8–10^. Using a classical twin study approach we and others have shown that human sensory performance is in large part genetically determined^11, 12^. Thus in healthy subjects up to 67% of the variation in vibration detection threshold sensitivity measured for a 125 Hz stimulus could be shown to be due to genetic factors^12^. Similarly high heritability was also found for cold and warm detection thresholds^12^. Quantitative sensory testing for pain modalities like heat pain threshold, acid induced pain and hyperalgesia have also been shown to exhibit high levels of heritability^11^. It is well known that single gene defects can, in rare cases, lead to a complete loss of pain sensation in man^13, 14^. More recently, it has been shown that single gene defects are also associated with loss and gain of function in human touch sensation^15–18^. For both pain and touch it is striking that mechanistic studies have revealed that often the genes associated with human sensory disorders code for ion channels involved in the transduction and transformation of sensory information at the peripheral endings of specific sensory neurons^15, 19–21^. It is clear that there are probably still many genes and gene variants that influence somatosensory performance whether the stimuli are tactile, thermal or painful.

**Figure 1:**
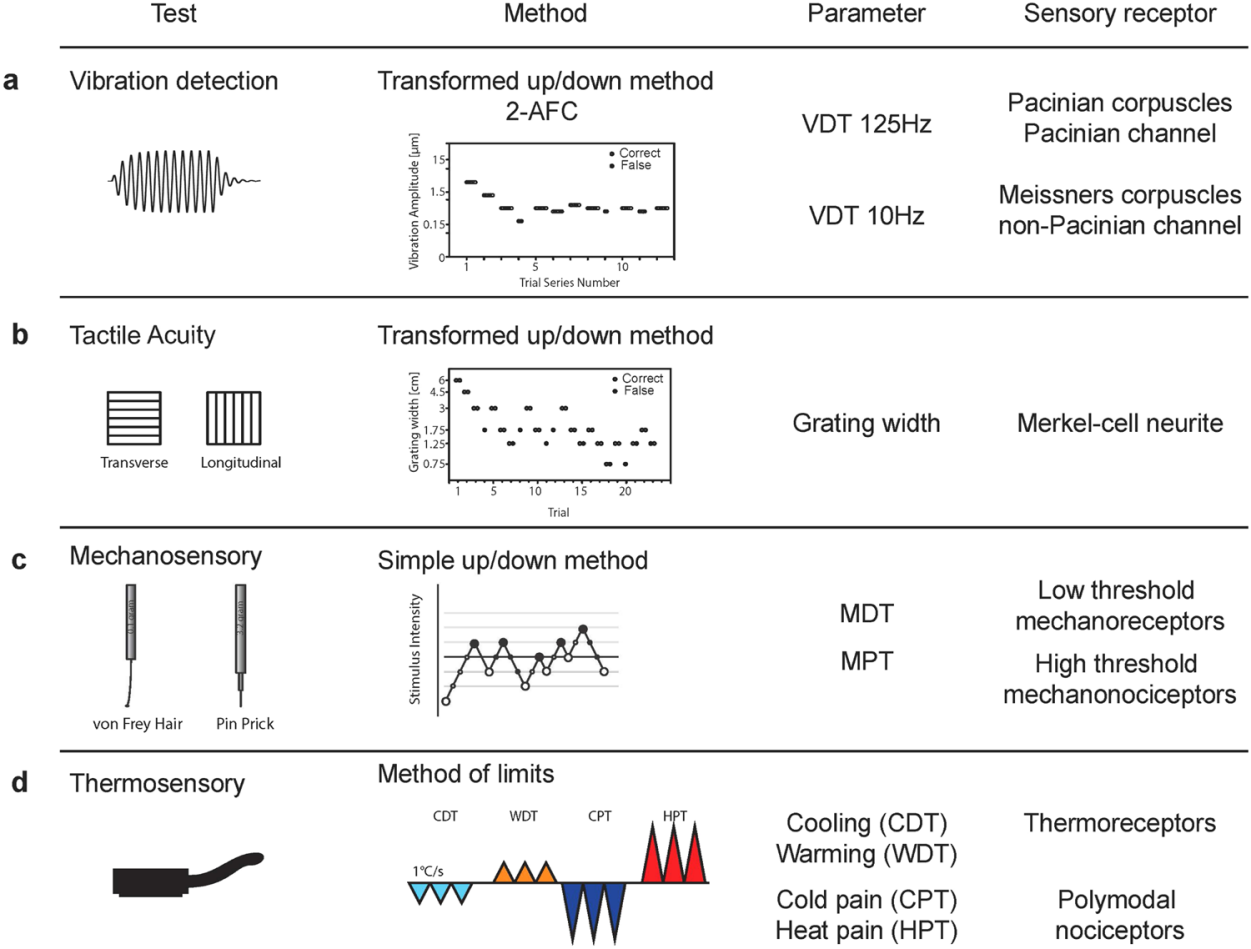
Overview of the sensory testing battery employed to generate the sensory profile for each tested subject. VDT, vibration detection threshold; MDT, mechanical detection threshold; MPT, mechanical pain threshold; CDT, cold detection threshold; WDT, warm detection threshold; CPT, cold pain threshold; HPT, heat pain threshold, 2-AFC; 2 alternative forced choice.

As well as genetic factors, human performance in quantitative sensory testing is also influenced by sex and importantly performance often decreases with age^3, 4, 12, 22^. We have shown previously that there are likely common genetic factors that influence hearing and touch^12^. Thus measurements of vibration detection thresholds and tactile acuity showed a correlation with hearing sensitivity and acuity^12^. In addition, children with congenital hearing impairment performed on average worse than controls with higher vibration detection thresholds and poorer tactile acuity^12^. In our previous study we did not make any measurements of pain sensitivity in hearing impaired children. Vibration detection thresholds in humans are known to be dependent on the frequency of the vibrotactile stimulus^6, 15^. In most studies a high frequency vibration is used (125 Hz) a stimulus that primarily probes sensations initiated by specialized rapidly-adapting mechanoreceptors that innervate Pacinian corpuscles^6, 18, 23^. In this study we also wished to probe tactile sensation mediated by mechanoreceptors that are selectively activated by low frequency vibration. Such receptors include rapidly-adapting mechanoreceptors innervating Meissner’s corpuscles in the glabrous skin, hair follicle afferents and slowly-adapting mechanoreceptors innervating Merkel cells^8, 10, 15^. Here we have used a broad battery of quantitative sensory tests, to probe variation in tactile, thermal and pain sensitivity in a highly homogenous population of adolescents (14–20 years old) (Fig. 1). We primarily asked whether children with congenital hearing impairments have altered somatosensory performance in specific sensory tasks compared to their age-matched controls.

## Results

One hundred and twenty-five healthy (125) and 39 hearing impaired participants, aged between 14 and 20 years, underwent quantitative sensory testing. Data obtained from 14 healthy participants were excluded due to incomplete testing (e.g. for logistical reasons not all tests in the battery completed), technical problems, or due to inconsistencies (e.g. high performance variability) during testing. Three of 39 hearing impaired subjects were excluded; one due to insufficient information on the probable cause of the hearing impairment and the other two due to a non-genetic origin of the hearing deficit. Therefore, data obtained from 111 healthy subjects (58 females and 53 males, mean age 16.0 and range 14–19 years) and 36 hearing impaired subjects (18 females and 18 males, mean age 16.7 and range 14–20 years) were included in the study. Thirty-one subjects from the hearing impaired cohort had non-syndromic hearing loss, 4 had Usher’s syndrome (3 with type 2 and one with type 1), and one Alport syndrome (probably autosomal recessive type). One hearing impaired subject had coeliac disease. Nine subjects had cochlear implants and 27 used hearing aids for moderate to severe hearing loss. Seven subjects had a positive family history of hearing loss. One hearing impaired subject reported difficulties perceiving wetness.

Psychophysical measures of sensory performance are often strongly influenced by age and gender^12, 24^. We controlled for age by selecting a young adolescent population with a narrow age range. Gender was controlled for by balancing the proportion of males to females in both groups. Comparison of psychophysical parameters between males and females in the control cohort revealed statistically significant differences in vibration detection thresholds (VDT) at 10 Hz but not at 125 Hz; mean VDT (mean ± SD, µm) for females 3.68 ± 1.48 versus 4.28 ± 1.57 in males (one-way ANOVA, F_(1,109)_ = 4.2, P = 0.04). Mechanical detection thresholds (MDT) were also significantly lower in female subjects compared to males (mean ± SD, mN; females: 1.1 ± 0.7, males 1.7 ± 0.9; one-way ANOVA, F_(1,109)_ = 16.2, P = 0.0001). Finally, warming detection thresholds were lower in control females (mean ± SD, Δ°C; females: 1.6 ± 0.4, males 1.8 ± 0.6; one-way ANOVA, F_(1,109)_ = 4.7, P = 0.03). There were no significant differences in performance between the two sexes for any of the other test parameters (Table 1).

**Table 1:** Analysis of variance, mean values and confidence intervals for psychophysical tests.

		Deaf		Control		ANOVA					
		Mean	95% CI	Mean	95% CI	Group Deaf/control		Gender Male/female		Interaction Group:Gender	
						F	P	F	P	F	P
VDT 10 Hz	µm	7.24	6.10–8.58	3.66	3.40–3.96	69.1	<0.001	5.3	<0.05	0.06	0.8
VDT 125 Hz	nm	119	95.0–149	96	87.0–105	4.6	<0.05	0.004	0.95	0.06	0.8
TA	mm	1.63	1.52–1.75	1.52	1.45–1.59	2.8	0.09	2.6	0.11	0.7	0.4
MDT	mN	1.31	1.10–1.57	1.13	1.01–1.26	1.9	0.17	16.8	<0.001	0.7	0.4
CDT	Δ°C	0.92	0.78–1.09	0.66	0.61–0.71	16.7	<0.001	2.9	0.09	0.2	0.6
WDT	Δ°C	1.62	1.46–1.80	1.62	1.54–1.71	0	0.99	6.4	<0.05	0.01	0.91
CPT	°C	13.4	10.2–16.7	11.79	10.1–13.4	0.9	0.34	1.02	0.31	0.9	0.3
HPT	°C	42.6	41.5–43.7	43.9	43.3–44.4	4.65	<0.05	1	0.31	0.9	0.3
MPT	mN	75.8	62.8–91.5	73.6	65.8–82.3	0.07	0.79	2.6	0.1	3.0	0.08

Mean values of VDT 10 Hz & 125 Hz, TAC, MDT, CDT, WDT, CPT, HPT, MPT were calculated by back transformation from the log-means. CI, confidence interval.

VDT 10 Hz and 125 Hz, vibration detection threshold at 10 Hz and 125 Hz; TA, tactile acuity; MDT, mechanical detection threshold; CDT cold detection threshold; WDT, warmth detection threshold; CPT, cold pain threshold; HPT, heat pain threshold; MPT, mechanical pain threshold.

**Figure 2:**
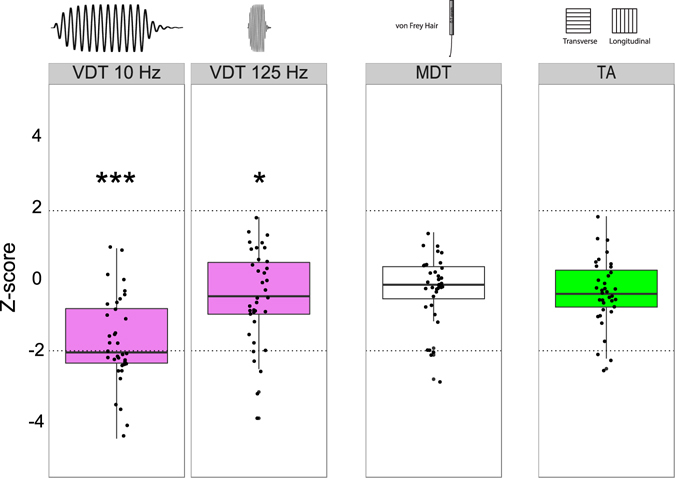
Vibration detection threshold (VDT) at 10 Hz and 125 Hz, mechanical detection threshold (MDT), and tactile acuity (TA) in the congenitally deaf cohort (n = 36). Each point represents the threshold for a single subject. All values are normalized for gender on a Z-scale. Dotted lines designate the upper (z = 1.96) and lower (z = −1.96) boundaries of the 95% confidence interval of the normal standard distribution of healthy subjects (n = 111). Z-scores > 0 indicate increased sensitivity. Z-scores < 0 indicate decreased sensitivity to sinusoidal vibrations. *P < 0.05, **P < 0.01, ***P < 0.001, unpaired T-test. Box plots characteristics: center lines show the medians; box limits indicate the 25th and 75th percentiles; whiskers extend 1.5 times the interquartile range from the 25th and 75th percentiles.

Hearing impaired subjects showed a robust and highly significant impairment in vibration detection ability compared to the control cohort (Table 1, Fig. 2). Vibration detection thresholds were significantly elevated for both the 10 and 125 Hz tasks, but the difference between the cohorts was largest for the 10 Hz task. The mean amplitude detected by hearing impaired subjects was shifted almost 2 fold to 7.24 µm compared to 3.66 µm in healthy controls and this was highly statistically significant (two-way ANOVA F_(1,143)_ = 68, P < 0.0001) (Fig. 2, and Table 1). Post-hoc tests revealed that both deaf males and females had higher vibration detection threshold than healthy males and females respectively (Post hoc Tukey test; P < 0.05). Since the performance of males and female control subjects in the 10 Hz test differ, we calculated z-scores for each male and female hearing impaired participant based on the mean and standard deviation of control males and females, respectively. Individual and mean z-scores for vibration detection threshold at 10 and 125 Hz (VDT), mechanical detection threshold and tactile acuity are presented in Fig. 2. There was no significant difference in mechanical detection threshold between deaf and healthy controls (Table 1). The average grating width orientation threshold measured with the tactile acuity cube test (TA) was calculated for each individual by averaging the thresholds measured for the index and little finger. There was no significant difference in tactile acuity measures between the control and hearing impaired cohorts (two-way ANOVA F_(1,143)_ = 2.7, P = 0.09). Nevertheless, there was a small subset of hearing impaired individuals with z-scores less than −1.96 indicating loss of function for this test (Fig. 2).

**Figure 3:**
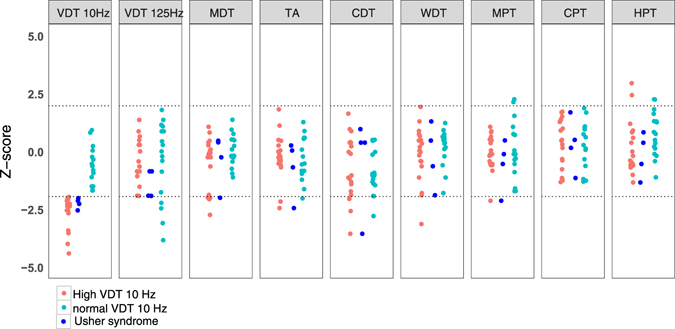
Dot plot of the z-score parameters in the congenitally deaf cohort. In red are deaf subjects with z-scores for VDTs at 10 Hz lower than −1.96. Blue represent the rest of the group. Participants with hearing impairment who had high threshold in VDT 10 Hz did not consistently have increased thresholds in other tests.

Eighteen of the hearing impaired participants had z-scores for VDT (10 Hz) below −1.96 (~2 standard deviations lower than the control mean; 50% of all tested subjects). The difference in VDT at 125 Hz between hearing impaired and control individuals was also statistically significant (two-way ANOVA F_(1,143)_ = 4.5, P = 0.03), but not large; the mean VDT was increased in hearing impaired individuals by ~25% (an absolute difference of just 23 nm, Table 1). However, it should be noted that the plot of VDT at 125 Hz shows 4 individuals with z-values that clearly reside in a loss of function window (below −1.96). Interestingly, the subject with the worst z-score for VDT at 125 Hz (z-score = −3.85) had self-reported difficulty in perceiving wetness. This subject did not otherwise exhibit extreme deficits in any of the other tests. We did not systematically ask subjects if they had difficulty perceiving wetness, and so it is unclear if this was the only such individual. Because the major effect was found for VDT at 10 Hz, we asked if study subjects with z-values lying in a loss of function window (below −1.96) also performed poorly in other tests. Hearing impaired subjects who performed poorly in the vibration detection task at 10 Hz did not perform poorly with the same test at 125 Hz. Neither did these individuals perform better or worse than the rest of the hearing impaired population in any of the other test batteries used (Fig. 3). Interestingly, hearing impaired individuals with an impairment in VDT tested at 125 Hz appear to be a different population of individuals than the majority of individuals with a VDT impairment at 10 Hz (Fig. 3). However, it can also be seen that the 4 subjects with Usher syndrome all had increased thresholds (z scores < −1.96) for 10 Hz vibrations (Fig. 3). The VDT at 125 Hz for these subjects were all below −1 z-unit but not below −1.96 which may indicate a moderate impairment that parallels the prominent deficits for 10 Hz vibration.

**Figure 4:**
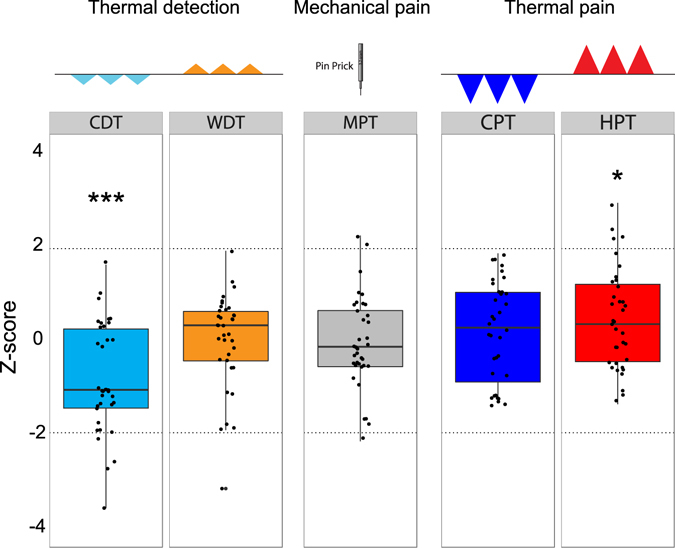
Sensory profiles in the hearing impaired cohort (n = 36). The z-values were normalized for gender on the z-scale. The z-scores for each deaf subject are represented as dot and box plots. Z-scores between 1.96 and −1.96 represent the normal range of healthy controls (n = 111). Z-scores > 0 indicate increased sensitivity to presented stimuli or in case of pain stimuli, lowered pain threshold. Z-scores < 0 indicate decreased sensitivity to presented stimuli, or in case of pain stimuli, higher pain threshold. *P < 0.05, **P < 0.01, ***P < 0.001, ANOVA. CDT, cold detection threshold; CPT, cold pain threshold; HPT, heat pain threshold; MDT, mechanical detection threshold; MPT, mechanical pain threshold; WDT, warm detection threshold.

Thermal detection thresholds for warming and cooling were also measured and we noted a strong and robust loss of function for cooling but not warm detection in the hearing impaired cohort (Table 1, Fig. 4). Thus the mean cold detection threshold was around 40% higher in the hearing impaired cohort compared to controls and this was statistically significant (two-way ANOVA F_(1,143)_ = 16.5, P < 0.0001). Post hoc Tukey tests revealed that the loss of cold detection was significant for hearing impaired male and females compared to controls (Post hoc Tukey test; P < 0.05). Examination of the distribution of z-scores for cold detection appeared to show two broad sub-groups: one group with z-values shifted to the loss-of-function direction and the other group with z scores around the control mean.

We measured several pain related psychophysical parameters in our cohorts including mechanical pain threshold and heat and cold pain thresholds. There was no indication that mechanical pain threshold differs between control and hearing impaired individuals (Fig. 4) with mean values in both groups being almost identical (Table 1). However, heat pain thresholds were significantly changed in hearing impaired individuals so that their thresholds were on average 1.3 °C lower than those found in controls (two-way ANOVA F_(1,143)_ = 4.7, P = 0.03). Four hearing impaired individuals had very low heat pain thresholds with z-scores that were above 1.96 (Fig. 4).

## Discussion

Quantitative sensory testing was used to systematically test whether congenital hearing impairment is associated with somatosensory deficits. In agreement with our previous study^12^, we could confirm in an independent patient cohort that many adolescents born with hearing impairment exhibit substantial deficits in touch sensation. The touch deficits we observed were most common and most severe for vibration detection at 10 Hz. Indeed 50% of the hearing impaired subjects (18/36) had vibration detection thresholds that were more than two standard deviations higher than the control mean (z-score > −1.96), and subjects with similarly impaired vibration detection thresholds at 125 Hz, but not at 10 Hz appeared to represent an additional population (4/36) thus at least 22/36 (61%) of hearing impaired subjects exhibit a severe vibration detection deficit. Thus distinct patient groups were found with deficits in vibration detection threshold at 125 Hz and 10 Hz, respectively. This finding strongly suggests that the underlying pathophysiology is specific for the function or connectivity of distinct sets of mechanoreceptors. Thus it is known that a 10 Hz vibration is primarily detected by rapidly-adapting Meissner’s corpuscle receptors and slowly-adapting Merkel cell mechanoreceptors^6, 25, 26^. In contrast, the detection of high frequency vibration of 125 Hz primarily requires rapidly-adapting mechanoreceptors that innervate Pacinian corpuscles^7, 18^. In this study we asked whether sensory deficits in subjects with hearing impairments extend to pain. We used a standardized testing protocol that included several elements of the QST of the German Research Network on Neuropathic Pain (DFNS)^2^. We found no clear deficits in pain sensitivity amongst hearing impaired individuals and this was especially clear for mechanical pain (Fig. 4). There was significantly increased heat pain sensitivity in the hearing impaired cohort, but this was a relatively small effect (Fig. 4). In addition to measures of tactile sensitivity, we also found a robust change in the ability of hearing impaired individuals to detect cooling, but no deficit was seen in warm detection (Table 1 Fig. 4). Several hearing impaired individuals (8/36) exhibited cold detection thresholds that were more than 1.5 standard deviations higher than the control means (z-score > −1.47). There appeared to be two groups of hearing impaired individuals those with cold detection thresholds clearly higher than the control mean and those with cold detection thresholds more similar to healthy controls. Indeed many of the hearing impaired participants with a z-score for CDT below −1.0 also had a z-score for vibration detection (10 or 125 Hz) that was below −1.5 (16/18 individuals). Cool objects have a distinct tactile quality compared to warm objects as exemplified by Weber’s effect, a cold coin feels heavier than a thermally neutral coin^27^. There is very recent evidence that information on cooling is processed by tactile responsive neurons in the primary somatosensory cortex^28^. Indeed, C-fiber polymodal nociceptors that are sensitive to cooling appear to provide information on non-noxious cooling directly to the somatosensory cortex^28^ and human polymodal C-fibers are also responsive to cooling^29^.

The study participants suffered hearing loss from birth and most of the cohort (31/36) had non-syndromic forms of hearing loss. We assume that the vast majority of the study participants suffered from deafness with a genetic cause. It is known that there are well over 100 genes that when mutated can cause non-syndromic sensorineural deafness^30^ and many of these genes are known to code for proteins that are essential for hair cell mechanotransduction^31^. We suggest that our data are consistent with the idea that a substantial number of gene mutations that lead to hearing loss also play a role in either the development or function of cutaneous mechanoreceptors. One surprising aspect of our study is how severe and specific the effects of congenital hearing loss are on touch sensation driven by afferents that are tuned to low frequency vibration. This suggests that many genes involved in hearing loss also have a role either in the development of rapidly-adapting and slowly-adapting mechanoreceptors or in modulating their functional properties or connectivity. The genetics of congenital hearing loss is highly complex as first, many genes are potentially involved and secondly, it is not uncommon that deafness is associated with mutations in two different hearing genes (compound heterozygotes)^32^. Thus even if we had detailed genetic information on our hearing impaired study participants, it might be difficult to link a specific type of mutation to the touch phenotype in a cohort this size. However, our study shows that by measuring vibration detection thresholds with a 10 Hz stimulus we are likely to identify a very substantial number of hearing impaired individuals with touch deficits. We tested a small number of patients with Usher syndrome and it is known that many Usher genes are directly involved in mechanotransduction in hair cells^33–36^. Here we found that the small number of Usher syndrome patients all had poor vibration detection thresholds especially at 10 Hz (Fig. 3). In our previous study we found an association between mutations in the USH2A gene (Usherin) and vibration detection performance at 125 Hz^12^. Our present data would suggest that using a 10 Hz vibration detection task may reveal more profound changes in touch sensitivity in patients with Usher syndrome.

Several smaller scale studies have investigated tactile perception in individuals with hearing impairment or individuals with deaf blindness^37–39^. In general these studies did not examine perceptual thresholds but rather frequency discrimination (using frequencies > 100 Hz), but in contrast to data on blind individuals^12, 37, 40^, there was little evidence that hearing impaired subjects perform better on tactile tests. There is good evidence that auditory and tactile information may be processed in common cortical areas^41, 42^, but interactions between auditory and tactile channels have been examined for high frequency channels ( > 100 Hz). It remains to be seen if there is significant cross-modality interactions for low frequency vibration which was most severely affected parameter in our hearing impaired cohort (Fig. 2). In general we would argue that hearing impairment in itself is unlikely to reduce vibration detection thresholds by a generalized negative effect on the development of cortical circuits. If a cortical development problem caused by hearing loss would underlie the loss of vibration sensitivity we would expect that all of our hearing impaired subjects exhibit clear touch deficits which they did not (Fig. 2).

In summary, in a well controlled study using young aged matched individuals we could show highly specific deficits in vibration sensitivity in hearing-impaired individuals. Interestingly, congenital hearing impairment was not associated with major changes in the detection of noxious mechanical stimuli. The fact that deficits were most common and penetrant for a 10 Hz vibration detection task suggest that hearing genes may have a role in the development, function, or connectivity of classical mechanoreceptors.

## Methods

The study was approved by the ethics committee of the Charité University Hospital. All experiments performed with human subjects were approved by Charité ethics committee and were in compliance with German and European union law.

### Study population

Two cohorts were studied one consisting of healthy high school children and one of high school children with congenital hearing loss. Adolescents were recruited from three Berlin high schools. Study participants were tested with a battery consisting of nine psychophysical tests applied to the skin. The hearing impaired cohort was recruited from the Margaretha-von-Witzleben School in Berlin (www.witzleben-schule.de). All subjects and their parents received written and oral information describing the study and signed an informed consent form. The subjects and schools were received compensation for taking part in the study. Because tactile sensitivity is known to change with age, we chose subjects with a narrow age range 14 to 20 years^24^. Eligible subjects were between 14 and 20 years of age, did not receive medication, and had no diabetes or hypertension. The following subject information was routinely documented: age, gender, handedness, history of ear infections, family history of hearing loss, hearing devices (hearing aids, cochlear implants), and comorbidities.

### Quantitative Sensory Testing

Subjects were seated comfortably in a quiet room at the school. Prior to testing, the participants were familiarized with the setup and their questions and concerns were addressed. Tests were completed in 2 sessions each of 45 minutes duration. Hearing impaired subjects were asked to switch off their hearing aids during the vibration detection threshold test. Sensory testing was done on the side of the dominant hand.

### Vibration detection threshold

The test has been previously described^43, 44^ (Fig. 1a). The device is composed of a linear Piezo actuator (P-602.1 L, Physik Instrument, Germany) whose displacements are driven and controlled by an amplifier/controller (E-665, Physik Instrument, Germany). Signals are processed by the PowerLab (PowerLab 4/35, ADInstruments, USA) data acquisition system. The vibration stimulus was 1.8 s long and the rise and fall time at on and offset were 500 and 600 ms, respectively, independent of the testing frequency or amplitude. The duration of the stimulus between the on and offset phases was 700 ms. A set of the vibration stimuli were employed that scaled logarithmically between 18 nm to 45 µm. For the 10 Hz and 125 Hz vibration test, the starting amplitude was set to 7.18 µm and 2.84, respectively. A monitor was employed to indicate the time interval at which the stimulus is presented, and a hand-held control (response box) allowed the subject to choose the interval at which a stimulus was perceived. The stimulating probe was made of a flat circular smoothed edge thermoplastic material attached to a screw head that is tightened directly to the moving part of piezoelectric actuator. The probe had a diameter of 8.21mm. The Piezo actuator with the probe is mounted on a balanced brass bar (weight 15.5 kg) that assures a resting force of 30 grams applied to the skin surface at the probe-skin contact. The subjects forearm was placed on a foam cushioned surface to prevent stray vibration. We used medical dough under the finger to ensure proper contact between the probe and the skin. The stimulating probe was placed on the small finger of the dominant hand just below the nail bed. The test protocol was delivered with a program scripted in LabChart software (Labchart 7, ADInstruments, USA) that implements the two-alternative forced choice (2-AFC) technique in the down/up staircase paradigm to estimate vibration detection threshold. A vibration stimulus was randomly presented only once during one of the two intervals, indicated visually to the subjects as “1” and “2” on a screen. After each trial the subject indicates with a button press whether the vibration was applied during the first or second test interval. The experimental session consisted of a series of trials at stimulus strengths that were chosen based on the transformed down-up staircase method. The experimental block ended after 8 reversal points were completed. The threshold is determined by calculating the median of the stimulus intensity of the last 6 reversals. This adaptive two-interval forced-choice procedure was used to obtain estimates of the detection threshold defined as the stimulus intensity at which the proportion of correct responses is 75%. Subject data was excluded from the results if performance inconsistencies occurred such as change in reversal point values higher than 4 amplitude levels.

### Tactile Acuity Test

Tactile acuity is determined with a two-alternative forced choice grating orientation test using the Tactile Acuity Cube (TAC) (MyNeurolab.com/Leica Microsystems) (Fig. 1b)^44, 45^. The 6 sides of the cube each contained a grating (bar and groove) whose widths are 0.75, 1.25, 1.75, 3.0, 4.5, and 6.0 mm. During the experiment the test subjects were blind-folded using shielded eyeglasses. The dominant hand is placed on a table with the palmar surface facing up. Starting with the widest width, the gratings of the Tactile Acuity Cube are applied in each trial for 2 s to the finger pad in one of the two orthogonal directions relative to the long axis of the finger (longitudinal or transverse), in a way that the cube exerts its whole weight on the finger (233 g). The subject indicated the perceived orientation of the alignment. The orientation of the cube was randomly selected by the experimenter. An adaptive method is used: a simple two-down (narrower grating when the subject answers correctly) and one-up (wider gratings when the subject answers incorrectly) procedure or algorithm is employed which converges to 71% correct response (the point at which the subject reliably perceives groove width). The test concluded after thirteen reversal points (perceptual deflection points) were determined and the median of the last 10 were taken as threshold. Thresholds were determined for the little finger and the index finger and the mean of the two values taken as tactile acuity.

### Mechanical detection threshold

Mechanical detection threshold was determined with a standardized set of 23 von Frey filaments (Optihair3-Set, Marstock Nervetest, Germany) (Fig. 1c). The filaments had uniform size and shape (round, diameter 0.5mm) and exerted discrete forces between 0.25–256 mN. The psychophysical method was a simple staircase algorithm (1 up and 1 down rule) that was completed after 5 reversals (change in tactile sensation). With the subject blindfolded with shielded glasses, the von Frey filaments were applied in an ascending order on the dorsal aspect of the hand for 1 second until the subject perceived a tactile sensation. The order was then reversed (descending) until the point where the subject did not perceive a tactile sensation. The threshold was calculated as the geometric mean force of the 5 reversals.

### Mechanical pain threshold

Mechanical pain threshold was tested with a set of 7 weighted pinprick stimulators (MRC systems, germany) (Fig. 1c). The tips have a diameter of 0.25 mm. The stimulators apply different forces between 8 and 512 mN. The psychophysical method was a simple staircase (1 up and 1 down rule) that was completed after 5 reversals that targeted the perception of sharpness that causes the feeling of pricking pain. The threshold was calculated as the geometric mean of the 5 reversal of pinprick intensities.

### Thermal detection and pain threshold

The thermal testing device, TSA II Thermosensory analyser (MEDOC, Israel), was employed to test for thermal detection and pain thresholds (Fig. 1d). The thermode had an area of 9 cm^2^ and cut off temperatures between 0 °C and 50 °C. The thermode heats up or cools down at a rate of 1 °C/s. All thresholds were determined using the method of limits. The thermode was placed on the volar aspect of the mid-forearm region. Three consecutive trials were done for each thermal sensation with 30 seconds interstimulus interval in the following order: cold detection, warm detection, cold pain and heat pain threshold. Study participants are asked to indicate at what point they start experiencing cooling, warming, cold and heat pain while the thermode is still in contact with the skin. The average thermode temperature of the 3 trials constitutes the above mentioned thermal threshold.

### Statistical analysis

The statistical analysis was done using R Software. Data were tested for normality and log-transformed when necessary before further statistical analysis except for raw data of CDT and HPT which were normally distributed. Test values were entered as dependent variables and group (hearing impaired and control) and gender (male, female) was entered as the independent variable. Differences in sensory parameter data between heraing and control groups were compared by a factorial analysis of variance (ANOVA) with the group (2 levels: hearing impaied, control) and gender (2 levels: male, female). Assumptions for error (normal distribution of residuals) and homogeneity of variance (Levene’s test) were determined before conducting the ANOVA. The factorial ANOVAs were obtained with type II sum of squares for sex to test for differences in means between males and females in the control data. The main effect for group was studied with type III method because the sample sizes were unequal. This was followed with Tukey posthoc tests if significant effects are detected. Z-transformations were utilized to compare single individual data profiles with the healthy control (HC) mean. Z-scores for each single parameter referenced to gender was calculated using the following expression: Z-score = (mean_Deaf_ − mean_HC_)/SD_HC_. The algebraic sign of z-score values for each parameter was adjusted so that it reflects the individual’s sensitivity for each parameter. Z-values above 0 indicate a gain of function, meaning the subject is more sensitive to the tested stimuli compared with HC; a z-value below 0 indicates as loss of function, meaning a reduced sensitivity. With this procedure sensory profiles could be generated where all the parameters had a standard normal distribution with a mean of 0 and standard deviation of 1 unit. Furthermore, individual z-scores lying outside of the 95% confidence interval (ie, z-score < 1.96 or > 1.96 standard deviation) of our HC can be identified. Correlation analysis was done with spearman’s correlation test. A *P* value of less than 0.05 was considered statistically significant.
